# Somatic Genetic Variation in Solid Pseudopapillary Tumor of the Pancreas by Whole Exome Sequencing

**DOI:** 10.3390/ijms18010081

**Published:** 2017-01-03

**Authors:** Meng Guo, Guopei Luo, Kaizhou Jin, Jiang Long, He Cheng, Yu Lu, Zhengshi Wang, Chao Yang, Jin Xu, Quanxing Ni, Xianjun Yu, Chen Liu

**Affiliations:** 1 Department of Pancreas Surgery, Fudan University Shanghai Cancer Center, Shanghai 200032, China; guomeng@fudanpci.org (M.G.); luoguopei@hotmail.com (G.L.); jinkaizhou@fudanpci.org (K.J.); longjiang@fudanpci.org (J.L.); Chenghe@fudanpci.org (H.C.); luyu@fudanpci.org (Y.L.); wangzhengshi@fudanpci.org (Z.W.); yangchao@fudanpci.org (C.Y.); xujin@fudanpci.org (J.X.); niquanxing@fudanpci.org (Q.N.); 2 Department of Oncology, Shanghai Medical College, Fudan University, Shanghai 200032, China; 3 Pancreatic Cancer Institute, Fudan University, Shanghai 200032, China

**Keywords:** SPT, exome sequencing, genetic variation, SNPs, indels

## Abstract

Solid pseudopapillary tumor of the pancreas (SPT) is a rare pancreatic disease with a unique clinical manifestation. Although *CTNNB1* gene mutations had been universally reported, genetic variation profiles of SPT are largely unidentified. We conducted whole exome sequencing in nine SPT patients to probe the SPT-specific insertions and deletions (indels) and single nucleotide polymorphisms (SNPs). In total, 54 SNPs and 41 indels of prominent variations were demonstrated through parallel exome sequencing. We detected that *CTNNB1* mutations presented throughout all patients studied (100%), and a higher count of SNPs was particularly detected in patients with older age, larger tumor, and metastatic disease. By aggregating 95 detected variation events and viewing the interconnections among each of the genes with variations, *CTNNB1* was identified as the core portion in the network, which might collaborate with other events such as variations of *USP9X*, *EP400*, *HTT*, *MED12*, and *PKD1* to regulate tumorigenesis. Pathway analysis showed that the events involved in other cancers had the potential to influence the progression of the SNPs count. Our study revealed an insight into the variation of the gene encoding region underlying solid-pseudopapillary neoplasm tumorigenesis. The detection of these variations might partly reflect the potential molecular mechanism.

## 1. Introduction

Solid pseudopapillary tumor (SPT, also known as solid pseudopapillary neoplasm) is an uncommon but distinct pancreatic tumor with a reported incidence of approximately 2% of all exocrine pancreatic neoplasms [1]. Most SPTs have been diagnosed in females with a mean age of 28 years [2,3], and have always presented characteristics of indolent biological behavior and high rates of long-term survival [1,2]. Surgical resection resulted in better outcome even in metastatic disease [4]. Although multiple studies have allowed insight into SNPs genetic pathogenesis, comprehensive exploration of the variations of the gene coding region has not been performed [4,5].

Variations of *KRAS*, *SMAD4*, *TP53* and *CDKN2A* have never been detected in SPT [5,6], which is different to the molecular changes seen in some malignancies such as pancreatic cancer. However, the significance of Wnt signaling with β-catenin mutations in SPT has been determined [4]. Almost all patients with SPT have mutations of the somatic β-catenin coding gene (*CTNNB1*), and numerous proteins associated with β-catenin have been detected as dysfunctional [5,7,8]. Normally, neoplasm development has been described as regulated by multiple events instead of a single key protein [9]. In SPT, other gene variations may have a synergistic effect on the biological behavior of the neoplasm.

In the present study, we applied whole exome sequencing to investigate the cause of the genetic variation of solid pseudopapillary tumor. By identifying the prominent variations of indels and SNPs, 95 events were detected which were observed to impact gene function. These events have enabled us to describe the potential molecular pathways involved in the pathogenesis of this disease.

## 2. Results

We performed whole-exome sequencing of paired SPT tissues from nine patients with SPT confirmed by pathology, including four males (aged from 26 to 51 years) and five females (aged from 25 to 43 years). All patients were diagnosed with pancreatic cystic, solid, or cystic-solid lesions. The clinical features of seven patients with non-metastatic disease and two patients with metastases are listed in Table 1. Each set of paired sequencing data from the neoplasm and adjacent tissues were compared to detect the SPT-specific gene variations.

### 2.1. Mononucleotide Variation in Solid Pseudopapillary Tumor of the Pancreas (SPT)

**Figure 1:**
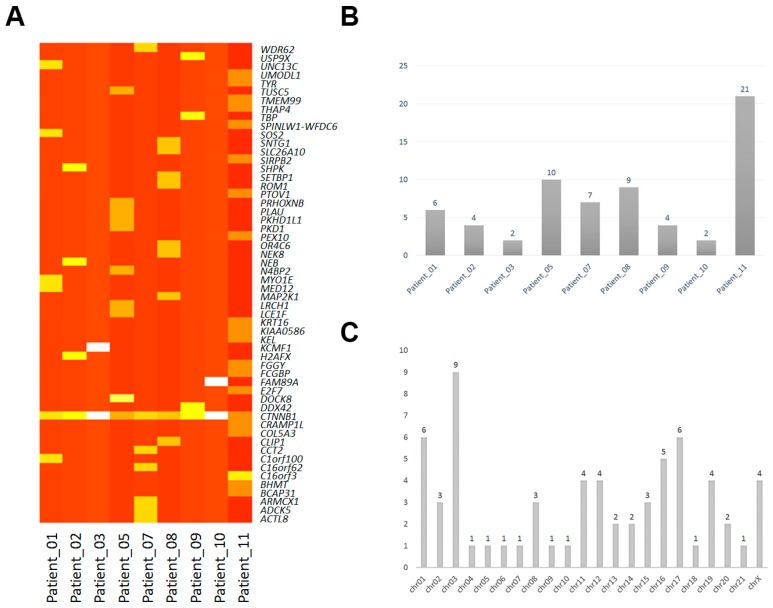
Single nucleotide polymorphism (SNP) distributions in solid pseudopapillary tumor of the pancreas. (**A**) The overview of non-synonymous mononucleotide variation corresponding to each samples. White and light yellow indicate the low and moderate variations count, respectively; Dark and brownish yellow indicate the multitude variations count, respectively; (**B**) SNP events distributed in each patient; (**C**) SNPs events distributed in each chromosome.

We performed an overview of all the non-synonymous mutations among the coding regions of each of the samples, and 65 prominent single base changes (SNPs) were detected (Table 2, Figure 1). The variations were detected in 56 genes, and *CTNNB1*, a β-catenin protein-coding gene, was found to be mutated in all the patients. In addition, no other general single sequence variation was found (Figure 1A). Almost all of the variations in the alleles were heterozygous mutations, and only one homozygous mutated base in the *MED12* gene had occurred (in patient number 1) (Table 2). Although the sample size investigated was limited, comparison of the incidence of SNPs between each case suggested that more SNPs events occurred in patients with distant metastases (*p* < 0.01) (Figure 1B). Interestingly, the patients with larger tumor size (diameter >100mm) had more SNPs detected than others with smaller size (Table 1, Figure 1B) (*p* < 0.01). In addition, the two patients with metastatic disease were older than the others. Moreover, analysis of the SNPs location showed that more mononucleotide variation was distributed in chromosomes 2, 1, and 17 (Figure 1C).

### 2.2. Insertions and Deletions in SPT

**Figure 2:**
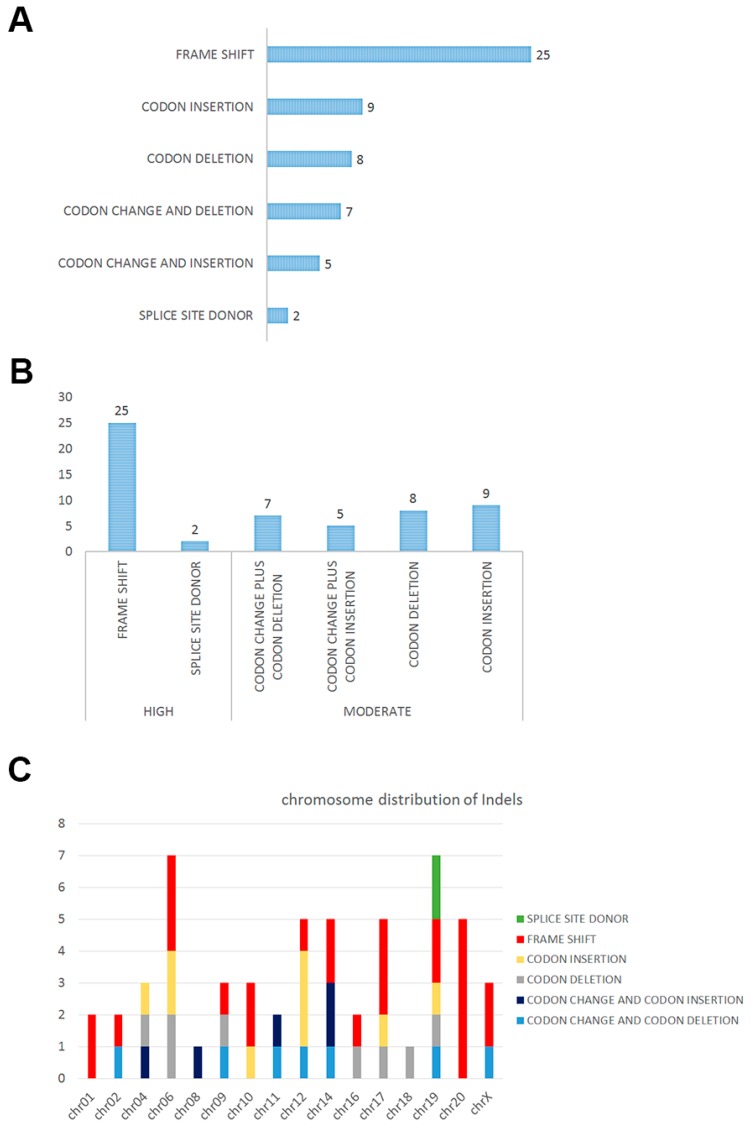
Functional annotation of indels detected in solid pseudopapillary tumor of the pancreas: (**A**) Indels would introduce frame shift, codon insertion, codon deletion, codon changes and deletion, codon changes and insertion and splicing alteration; (**B**) high and moderate impact of each indels by predicting; and (**C**) indels with different impact depth distributed in chromosomes.

In total, 56 significant insertions and deletions (indels) in the DNA were detected in the nine subjects, and 41 known genes were associated with those indels (Table 3). Functional annotation showed that a number of (25 of 56) indels would introduce a frame shift, and two indels would generate a splicing alteration (Figure 1A). We predicted that the impact of these sequence changes, 27 indels showing frame shift and/or splicing site changes, might be important in the biological activity of the cell, and that the other 29 events might play secondary roles (Figure 2B). Chromosome distribution showed that the regions of high impact were mostly located in chromosomes 19 and 20 (Figure 2C). We also compared the genes involved in indels and SNPs, and only one common gene, TBP (TATA-box binding protein), was detected.

### 2.3. The Network of Indels and Single Nucleotide Polymorphisms (SNPs) Related Genes

**Figure 3:**
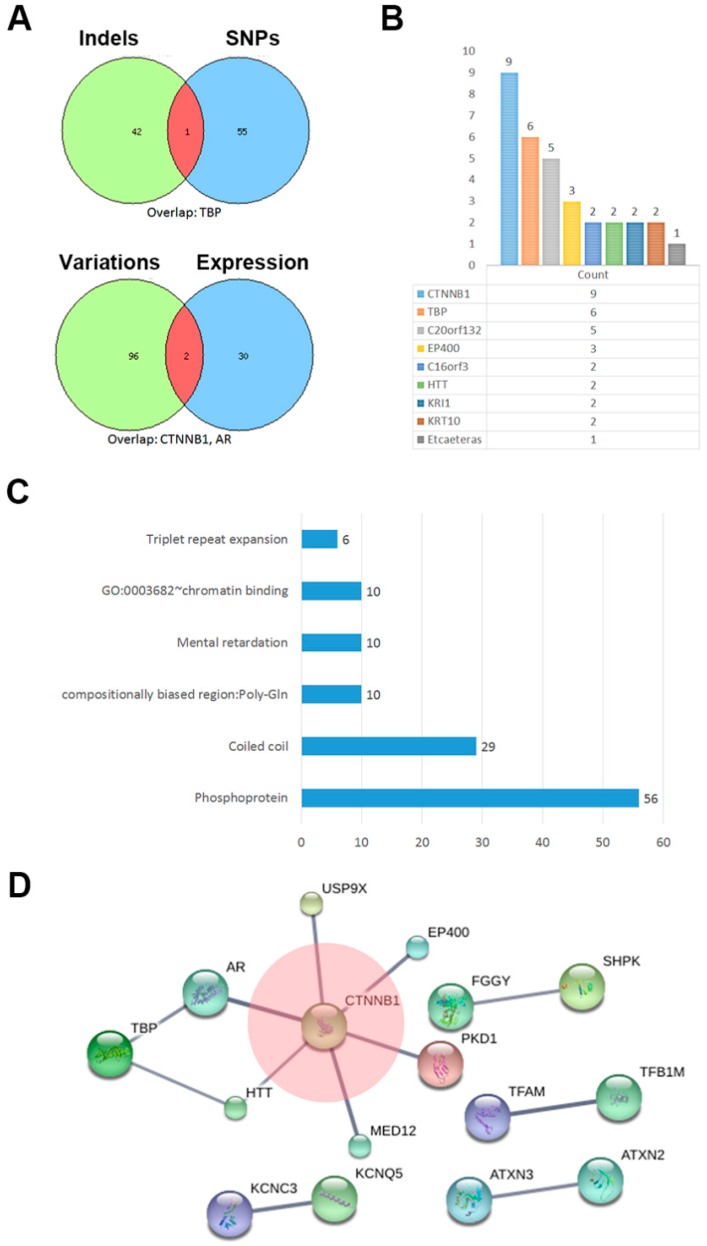
Combined set of variated genes: (**A**) Comparison of indels with SNPs involved genes (**top**) and present combined set with previously reported abnormally expressed genes (**bottom**) in SPN; (**B**) the variation events count of each homologous gene; (**C**) functions and pathways enrichment of combined variation events; and (**D**) network analysis according to String database.

In neoplasm progression, indels and SNPs cause gene functional variation [10], and gene expression also regulates important cellular activities. We compared the combined set of gene variations with previously reported abnormally expressed genes [5] in SPT, and the results showed an overlap of two genes, *CTNNB1* and *AR* (Figure 3A, bottom Venny schedule). Additionally, *CTNNB1* had the highest rate of variation events in the combined set. (Figure 3B). Phosphoproteins was shown as the biggest cluster based on the functions and pathway correlations (Figure 3C). Details of each cluster are listed in Table 4.

According to annotating protein–protein interaction using String database, *CTNNB1* was shown as a hub and directly connected with another six genes with a high confidence (score > 0.90) (Figure 3D). PKD1 (Protein Kinase D1), a serine-threonine kinase, has been reported to modulate the β-catenin functions in colon cancer [11]. The deubiquitination protein USP9X was shown to be required for lymphocyte activation [12]. EP400 is an E1A binding protein and deposits the histone variant H3.3 into chromatin alongside histone H2AZ and contributes to gene regulation [13]. The *HTT* gene coding for Huntington protein, is mutated in Huntington’s disease but is ubiquitously expressed, and mutant *HTT* also influences cancer progression [14]. Additionally, other protein–protein connections such as KCNC3 vs. KCNQ5, ATXN3 vs. ATXN2, TFAM vs. TFB1M, and FGGY vs. SHPK also showed stronger paired connections.

## 3. Discussion

The low incidence of solid pseudopapillary tumor of the pancreas determined that large-scale susceptibility gene screening was unachievable. To explore the potential pathogenic gene, we describe here the first paired whole genome sequencing of SPT in the Chinese population with a limited sample size (nine neoplasm tissues vs. nine adjunct tissues). Our data revealed that multiple protein-coding related variations participated in SPT disease progression. However, gene variation distributions in each case are widely divergent. Even though *CTNNB1* mutations were detected throughout all patients, the mutated nucleic acid sites were different (Table 2). Those diversified variations suggested that SPT is a multi-heterogeneity disease, which might be caused by the dysregulation in the development of pancreas.

The function network suggests that *CTNNB1* may work as a hub and be closely connected with other gene variations, such as *USP9X*, *EP400*, *PDK1*, *MED12*, *HTT* and *AR*. Some of those genes have been reported to play an important role in other cancers [15,16,17]. Both indels and SNP sets showed TBP (TATA-box binding) dysfunction (Figure 1A), and this might cause the hallmarks of oncogene-induced replication stress, including replication fork slowing, DNA damage, and senescence [18]. Comparing similarities of gene abnormality with former expression data [5], we noticed that CTNNB1 and AR (androgen receptor) were in the intersection, suggesting that AR signaling was also closely related [19]. Most colon cancer development and progression is involved in dysregulation of the β-catenin signaling pathway, and PKD1 was previously reported to directly interact with β-catenin, and to attenuate β-catenin transcriptional activity by decreasing nuclear β-catenin levels, which eventually suppressed colon cancer growth [11].

As the core portion in the co-connected network and the focus of multiple studies, the Wnt/β-catenin (*CTNNB1* coding) pathway played an important role to facilitate carcinogenesis through regulated or unregulated changes in gene transcription [4,7,20,21]. Although considerable detail had revealed that the upstream factors induced activation of β-catenin in the cytoplasm, the mechanism by which β-catenin is involved with connected gene variations in different neoplasms is much less known [22]. In this study, we detected that β-catenin was mutated in all neoplasms studied (100%), and that this frequency was higher than previously reported (approximate 90%), suggesting that *CTNNB1* mutation is ubiquitous in SPT patients. The detection level may depend on detection methods. Moreover, functionally associating or physically binding with other candidates indicated that the effect of β-catenin might require assistant factors [15,23].

Among the studied patients, only patients 5 and 11 showed a distant metastasis phenotype. We detected many more SNPs were distributed in the metastatic disease compared to non-metastatic cases. Although this interesting phenomenon requires extended study, it suggests that enriched mutation might accelerate metastatic disease. Analogously, enriched mutation was also potentially related to larger tumor size. These discoveries have not been reported previously.

Based on functional annotations of indels adding SNPs genes, phosphoproteins were shown as the biggest cluster, revealing that most protein variations participated in signaling transduction. For instance, USP9x, a deubiquitinase, and connected with CTNNB1 as shown in the network, has been reported to be required for PKCβ kinase activity and induced the cell survival and tumor-promoting activities of Notch signaling in cancer [12,24]. Additionally, significant enrichment of candidates also indicates an involvement in coiled-coil protein and mental retardation, suggesting the variation might cause structural abnormality and nervous system metastasis. Distinguishing with most previously study, we investigated the broad spectrum genes variations in SPT. All detected genetic variations need to be further verified.

## 4. Materials and Methods

### 4.1. Patients and Tissues

Eleven patients diagnosed with SPT and who underwent radical surgery in Shanghai Cancer Center, Fudan University (Shanghai, China), between 2010 and 2014 were selected for this study. SPT is circumscribed, solid, cystic masses and the pathology microscopic characteristic is typical pseudopapillae composed of central fibrovascular stalks embosomed by discohesive tumor cells with monotonous nuclei, absent nuclear pleomorphism, and low mitotic activity (Figure S1) [25,26]. The diagnoses of the resected tissues were confirmed by the Department of Pathology and 2 specimens (patient number 4 and patient number 6) were excluded because of the limited content of tumor cells (<30%). Clinical information regarding patient age, gender, TNM stage, tumor size, tumor location, and metastatic of non-metastatic disease, were collected from medical record files. TNM staging of each patient was based on AJCC (American Joint Committee on Cancer) classification criterion. Paired carcinoma and adjacent tissue specimens from the patients were frozen in liquid nitrogen and then store at −80 °C. The study was approved by the ethics committee of Fudan University Shanghai Cancer Center (ethical approval number: 050432-4-1212B, ethical approval date: 24 December 2012). Before the project began, written informed consent from all 9 patients was obtained, and the clinical events were evaluated based on the original histopathology reports and clinical records.

### 4.2. DNA Extraction and Exome Sequencing

Before DNA Extraction, frozen sections of each tissue were stained with H&E to ensure the tumor cell number was more than 25% in the tissue. Genomic DNA from 9 tissues from each patient was extracted using a DNeasy Blood & Tissue Kit (Qiagen, Hilden, Germany). The exome of the genomic DNA was captured and sequenced using Agilent SureSelect system (BGI Co., Shenzhen, China) according to the manual. The DNA sample of genomic was fragmented randomly. The 150- to 200-bp fragments was utilized for the library and the adaptors were subsequently ligated to the fragments at both ends. The adapter-ligated templates were purified according to Agencourt AMPure SPRI beads (Beckmancoulter, Brea, CA, USA). For enrichment, the extracted DNA was amplified by LM-PCR (ligation-mediated PCR), purified, and hybridized to the SureSelect Biotinylated RNA Library (BAITS) (ABI, Waltham, MA, USA). After 24 h incubation, hybridized fragments were bound to the streptavidin beads whereas non-hybridized fragments were washed out. To estimate the magnitude of enrichment, captured LM-PCR products were analyzed by Agilent 2100 Bioanalyzer (Agilent Technologies, San Jose, CA, USA). Subsequently, the captured library was loaded on a Hiseq2000 platform (Illumina, San Diego, CA, USA) and sequenced in high-throughput with depth of more than 100× to ensure that each sample met the desired average sequencing depth. Raw image files were processed by Illumina basecalling Software 1.7 (Illumina) and the sequences information were generated as 90/100 bp pair-end reads. Representative variations of SNPs and indels were subsequently validated by Sanger sequencing (Figure S2).

### 4.3. Read Mapping and Standard Bioinformatics Analysis

The sequencing data (raw data) generated from the Illumina software (Illumina basecalling Software 1.7) was needed to conduct cleaning and mapping. The adapter sequence in the raw data and low quality sequences which had too many unknown bases or low base quality were excluded. Clean data was produced and aligned by BWA (http://biobwa.sourceforge.net/) and formatted the sequence into binary BAM files. The BAM format files were established mate information of the alignment, added read group information and removed duplicate reads caused by PCR. Clean reads were processed by mapped to the reference human genome (GRCh37/hg19) from UCSC database (http://genome.ucsc.edu/) using SOAPalinger (http://soap.genomics.org.cn/index.html). Single Nucleotide Polymorphisms (SNPs) were detected according to SOAPsnp (http://soap.genomics.org.cn/soapsnp.html). Indels were aligned to the reference human genome from UCSC using BWA and further conduct with the Genome Analysis Toolkit (GATK v1.6) for recalling. Variants in the non-coding region and synonymous mutations were removed. SNPs and indels with higher frequency (>0.5%) noted in dbSNP (http://www.ncbi.nlm.nih.gov/projects/SNP/), 1000 Genomes (ftp://www.1000genome.org), HapMap were also filtered out. Quality Control (QC) was processed in the steps of the clean data, the alignment, and the identified variant.

### 4.4. Exome Homozygosity Mapping

Large stretches of the homozygous region were detected using the whole genome sequencing data. As markers to create a genetic map, all the autosomal dbSNP sites and novel SNPs that had ≥20-fold coverage of the exome target regions were examined. For the homozygous markers selection, variants with ≥95% of all reads displaying an identical SNP allele and covering at least 5-fold of the region were taken into consideration; for heterozygous markers selection, SNPs with 30% to 70% of all variation reads and which covered at least 10-fold were taken into consideration. The other SNPs with <30% or with 70%–95% variation reads were considered ambiguous. Perl script was utilized for statistical analysis of the distribution of map markers along the genome. A window of 500 markers, containing a maximum of 2 heterozygous markers and allowing a maximum gap between 2 adjacent markers of 500 KB, was adopted. A homozygous stretch by coalescence of all qualified windows with a minimum of 1 MB in length identified as a genomic region.

### 4.5. Cluster and Network

Each of the genes detected in the neoplasms with prominent SNPs and indels was functionally annotated and clustered by David database (https://david.ncifcrf.gov). After importing the genes list into String database (http://www.string-db.org), the high confidence (>0.7) connection between genes was presented, which might be co-mentioned or mutually bound.

### 4.6. Statistical Analysis

Statistical analysis was performed using SPSS software (v13.0, Chicago, IL, USA). Data were analyzed and statistically assessed by Fisher’s exact tests. *p* < 0.01 was considered to be significant for all statistical analyses.

## 5. Conclusions

In the current study, we conducted whole exome sequencing in 9 SPT patients, which detected 54 SNPs and 41 indels of prominent variations in total. Multiple SNPs with a higher count was found to correlate with adverse clinical manifestations. In addition to be detected throughout all cases, *CTNNB1* mutation was presented to potentially collaborate with other gene variations. The aberration events involved in other cancers also showed the potential to stimulate the progression of SPT. This work revealed an insight into the variation of the gene encoding regions might partly reflect the potential molecular mechanism of SPT.

## Figures and Tables

**Table 1:** Clinicopathological characteristics of patients.

Patients	Gender	Age (Years)	Size (mm)	TNM Stage	Location	Distant Metastasis (Yes/No)	CA19-9 Value	Surgical Procedures
1	male	35	18	I	head	No	no abnormal	distal pancreatectomy
2	male	33	50	II	body and tail	No	no abnormal	distal pancreatectomy
3	male	26	70	II	body and tail	No	no abnormal	distal pancreatectomy
5	female	43	108	II	head	Yes	no abnormal	total pancreatectomy
7	female	30	45	II	body and tail	No	no abnormal	distal pancreatectomy
8	female	31	45	II	head	No	no abnormal	pancreaticoduodenectomy
9	female	25	50	II	body and tail	No	no abnormal	distal pancreatectomy
10	female	25	NA	II	head	No	no abnormal	pancreaticoduodenectomy
11	male	51	138	IV	body and tail	Yes	no abnormal	distal pancreatectomy

**Table 2:** Information of prominent SNPs in each patient.

Samples	Gene	Biotype	Transcript	Codon	Chromosome	Alleles
Patient_01	*C1orf100*	Missense	NM_001012970:p.Tyr78Cys	tAt/tGt	chr01	het
	*CTNNB1*	Missense	NM_001098209:p.Asp32Tyr	Gac/Tac	chr03	het
	*MED12*	Missense	NM_005120:p.Arg1295Cys	Cgt/Tgt	chrX	hom
	*MYO1E*	Missense	NM_004998:p.Ser179Arg	agT/agG	chr15	het
	*SOS2*	Missense	NM_006939:p.Leu793Ile	Ctt/Att	chr14	het
	*UNC13C*	Missense	NM_001080534:p.Lys1395Met	aAg/aTg	chr15	het
Patient_02	*CTNNB1*	Missense	NM_001098209:p.Asp32Gly	gAc/gGc	chr03	het
	*H2AFX*	Missense	NM_002105:p.Leu98Arg	cTg/cGg	chr11	het
	*NEB*	Missense	NM_001164507:p.Asp5797Asn	Gat/Aat	chr02	het
	*SHPK*	Missense	NM_013276:p.Glu477Asp	gaA/gaC	chr17	het
Patient_03	*CTNNB1*	Missense	NM_001098209:p.Gly34Arg	Gga/Aga	chr03	het
	*KCMF1*	Missense	NM_020122:p.Arg257His	cGt/cAt	chr02	het
Patient_05	*CTNNB1*	Missense	NM_001098209:p.Ser37Pro	Tct/Cct	chr03	het
	*DOCK8*	Missense	NM_203447:p.Val245Met	Gtg/Atg	chr09	het
	*LCE1F*	Missense	NM_178354:p.Arg83His	cGt/cAt	chr01	het
	*LRCH1*	Missense	NM_001164211:p.His745Arg	cAt/cGt	chr13	het
	*N4BP2*	Missense	NM_018177:p.Thr92Ile	aCc/aTc	chr04	het
	*PKD1*	Missense	NM_001009944:p.Arg4249Cys	Cgc/Tgc	chr16	het
	*PKHD1L1*	Missense	NM_177531:p.Ile2532Ser	aTt/aGt	chr08	het
	*PLAU*	Missense	NM_002658:p.His224Gln	caC/caG	chr10	het
	*PRHOXNB*	Missense	NM_001105577:p.Gly116Arg	Ggt/Cgt	chr13	het
	*TUSC5*	Missense	NM_172367:p.Ser93Thr	Tcc/Acc	chr17	het
Patient_07	*ACTL8*	Missense	NM_030812:p.Arg48His	cGt/cAt	chr01	het
	*ADCK5*	Missense	NM_174922:p.Arg449His	cGc/cAc	chr08	het
	*ARMCX1*	Missense	NM_016608:p.Cys144Tyr	tGc/tAc	chrX	het
	*C16orf62*	Missense	NM_020314:p.Ala53Glu	gCg/gAg	chr16	het
	*CCT2*	Missense	NM_006431:p.Gly98Asp	gGc/gAc	chr12	het
	*CTNNB1*	Missense	NM_001098209:p.Ser33Cys	tCt/tGt	chr03	het
	*WDR62*	Missense	NM_001083961:p.Val407Ile	Gtt/Att	chr19	het
Patient_08	*CLIP1*	Missense	NM_001247997:p.Ile450Val	Att/Gtt	chr12	het
	*CTNNB1*	Missense	NM_001098209:p.Ser37Phe	tCt/tTt	chr03	het
	*MAP2K1*	Missense	NM_002755:p.Leu42His	cTt/cAt	chr15	het
	*NEK8*	Missense	NM_178170:p.Asp530Asn	Gac/Aac	chr17	het
	*OR4C6*	Missense	NM_001004704:p.Phe104Ser	tTc/tCc	chr11	het
	*ROM1*	Missense	NM_000327:p.Ala265Glu	gCa/gAa	chr11	het
	*SETBP1*	Missense	NM_015559:p.Tyr1327Cys	tAt/tGt	chr18	het
	*SLC26A10*	Missense	NM_133489:p.Val488Met	Gtg/Atg	chr12	het
	*SNTG1*	Missense	NM_018967:p.Arg202Gln	cGa/cAa	chr08	het
Patient_09	*CTNNB1*	Missense	NM_001098209:p.Ser37Phe	tCt/tTt	chr03	het
	*DDX42*	Missense	NM_007372:p.Thr581Ala	Acc/Gcc	chr17	het
	*TBP*	Missense	NM_003194:p.Thr106Ala	Acg/Gcg	chr06	het
	*USP9X*	Missense	NM_001039590:p.Asn2098Ser	aAt/aGt	chrX	het
Patient_10	*CTNNB1*	Missense	NM_001098209:p.Ser33Phe	tCt/tTt	chr03	het
	*FAM89A*	Missense	NM_198552:p.Ser175Cys	tCc/tGc	chr01	het
Patient_11	*BCAP31*	Missense	NM_001139457:p.Ile190Val	Att/Gtt	chrX	het
	*BHMT*	Missense	NM_001713:p.Asp105Asn	Gac/Aac	chr05	het
	*C16orf3*	Missense	NM_001214:p.Val60Ile	Gta/Ata	chr16	het
	*C16orf3*	Missense	NM_001214:p.Ser57Gly	Agc/Ggc	chr16	het
	*COL5A3*	Missense	NM_015719:p.Gly533Val	gGa/gTa	chr19	het
	*CRAMP1L*	Missense	NM_020825:p.Pro818Thr	Ccc/Acc	chr16	het
	*CTNNB1*	Missense	NM_001098209:p.Asp32Tyr	Gac/Tac	chr03	het
	*E2F7*	Missense	NM_203394:p.Phe873Val	Ttt/Gtt	chr12	het
	*FCGBP*	Missense	NM_003890:p.Gly4778Asp	gGc/gAc	chr19	het
	*FGGY*	Missense	NM_001113411:p.Ser21Asn	aGt/aAt	chr01	het
	*KEL*	Missense	NM_000420:p.Arg393Gln	cGg/cAg	chr07	het
	*KIAA0586*	Missense	NM_001244189:p.Lys953Ile	aAa/aTa	chr14	het
	*KRT16*	Missense	NM_005557:p.Gly69Cys	Ggc/Tgc	chr17	het
	*PEX10*	Missense	NM_153818:p.Leu221His	cTc/cAc	chr01	het
	*PTOV1*	Missense	NM_017432:p.Lys212Met	aAg/aTg	chr19	het
	*SIRPB2*	Missense	NM_001122962:p.Gly94Arg	Ggg/Agg	chr20	het
	*SPINLW1-WFDC6*	Missense	NM_001198986:p.Glu141Lys	Gaa/Aaa	chr20	het
	*THAP4*	Missense	NM_015963:p.Gly111Ser	Ggt/Agt	chr02	het
	*TMEM99*	Missense	NM_001195386:p.Asp195Asn	Gac/Aac	chr17	het
	*TYR*	Missense	NM_000372:p.Thr292Met	aCg/aTg	chr11	het
	*UMODL1*	Missense	NM_173568:p.Asp814Glu	gaC/gaG	chr21	het

Codon: Capital letter represents the variational base and lowercase represents the uniformity.

**Table 3:** Impact and functional annotations of detected Indel variations.

Impact	Function	Chr	Gene	Reference	Observation	Alleles
High	FS	chr01	*AHDC1*	T	TG	het
High	FS	chr01	*LRRIQ3*	G	GT	het
High	FS	chr10	*NOC3L*	AT	A	het
High	FS	chr10	*TFAM*	CA	C	het
High	FS	chr12	*TDG*	G	GA	het
High	FS	chr14	*CCNK*	G	GC	het
High	FS	chr14	*PAPOLA*	TG	T	het
High	FS	chr16	*IRX5*	AGG	A	het
High	FS	chr17	*ACSF2*	T	TAA	het
High	FS	chr17	*KRT10*	CCGCCG	C	het
High	FS	chr17	*KRT10*	TG	T	het
High	FS	chr19	*CAPN12*	G	GC	het
High	FS	chr19	*KCNC3*	C	CG	het
High	FS	chr02	*SNED1*	A	AC	het
High	FS	chr20	*C20orf132*	GACCT	G	het
High	FS	chr20	*C20orf132*	GC	G	het
High	FS	chr20	*C20orf132*	GAGGAGTT	G	het
High	FS	chr20	*C20orf132*	CG	C	het
High	FS	chr20	*C20orf132*	TGG	T	het
High	FS	chr06	*TBP*	AGC	A	het
High	FS	chr06	*TBP*	AG	A	het
High	FS	chr06	*TFB1M*	CAA	C	het
High	FS	chr09	*PHF2*	A	AG	het
High	FS	chrX	*PLXNA3*	T	TG	het
High	FS	chrX	*RBM10*	CA	C	het
High	SSD	chr19	*KRI1*	CCATCA	C	het
High	SSD	chr19	*KRI1*	CCATCA	C	het
Moderate	C & D	chr11	*SCUBE2*	GGCA	G	het
Moderate	C & D	chr12	*ATXN2*	GGCT	G	het
Moderate	C & D	chr14	*MAP3K9*	GCCT	G	het
Moderate	C & D	chr19	*SAFB2*	GTAC	G	het
Moderate	C & D	chr02	*GIGYF2*	CACA	C	het
Moderate	C & D	chr09	*TPRN*	TTCC	T	het
Moderate	C & D	chrX	*AR*	AAGAGACTAGCCCCAG	A	het
Moderate	C & I	chr11	*KRTAP5-8*	T	TCCG	het
Moderate	C & I	chr14	*ATXN3*	C	CCTG	het
Moderate	C & I	chr14	*IRF2BPL*	C	CTGCTGT	het
Moderate	C & I	chr04	*HTT*	A	AACAGCC	het
Moderate	C & I	chr08	*ATAD2*	A	ATCG	het
Moderate	CD	chr16	*APOBR*	TGGGACAGCCTCAGGAGGGGAGGAGGCC	T	het
Moderate	CD	chr17	*KDM6B*	TCAC	T	het
Moderate	CD	chr18	*MBD2*	CGCA	C	het
Moderate	CD	chr19	*ARID3A*	GGGA	G	het
Moderate	CD	chr04	*ADAM29*	GTGACACCCTCCCAGAGGCAACCTCAGT	G	het
Moderate	CD	chr06	*KCNQ5*	AGCG	A	het
Moderate	CD	chr06	*TBP*	GCAA	G	het
Moderate	CD	chr09	*RNF20*	TGTTGACTCTGAAGACTCA	T	het
Moderate	CI	chr10	*C10orf140*	C	CCCTCCT	het
Moderate	CI	chr12	*EP400*	A	ACAG	het
Moderate	CI	chr12	*EP400*	A	ACAG	het
Moderate	CI	chr12	*EP400*	G	GCAA	het
Moderate	CI	chr17	*KRTAP4-5*	T	TGGCAGCAGCTGGGGC	het
Moderate	CI	chr19	*ZNF814*	C	CATA	het
Moderate	CI	chr04	*HTT*	A	ACCGCCGCCG	het
Moderate	CI	chr06	*TBP*	A	ACAG	het
Moderate	CI	chr06	*TBP*	A	ACAG	het

FS: frame shift, SSD: splice site donor, CD: codon deletion, CI: codon insertion, G & I: codon change plus codon insertion, G & D: codon change plus codon deletion, Chr: chromosome.

**Table 4:** Ontology terms and annotations of indels adding SNPs genes.

Category	Term	Count	Genes	Benjamin	FDR
Up keywords	Phosphoprotein	56	*PLXNA3*, *KCNC3*, *TUSC5*, *E2F7*, *CCT2*, *CTNNB1*, *KCNQ5*, *MAP3K9*, *H2AFX*, *RBM10*, *AR*, *CCNK*, *C16ORF62*, *MED12*, *KRT10*, *KIAA0586*, *MBD2*, *LRCH1*, *KRT16*, *BHMT*, *NEK8*, *CLIP1*, *UNC13C*, *RNF20*, *GIGYF2*, *EP400*, *KDM6B*, *PTOV1*, *IRX5*, *THAP4*, *KEL*, *USP9X*, *NOC3L*, *N4BP2*, *TFAM*, *APOBR*, *PKD1*, *KRI1*, *DDX42*, *MAP2K1*, *HTT*, *MYO1E*, *ARID3A*, *ATAD2*, *DOCK8*, *SAFB2*, *ATXN2*, *ATXN3*, *PAPOLA*, *PHF2*, *KCMF1*, *WDR62*, *IRF2BPL*, *PLAU*, *TPRN*, *AHDC1*	0.004376	0.086018
Up keywords	Coiled coil	29	*LRRIQ3*, *THAP4*, *NOC3L*, *TBP*, *N4BP2*, *MAP3K9*, *PKD1*, *KRI1*, *DDX42*, *AR*, *MAP2K1*, *HTT*, *ATAD2*, *KRT10*, *KIAA0586*, *BCAP31*, *ATXN2*, *ATXN3*, *PHF2*, *KCMF1*, *LRCH1*, *IRF2BPL*, *KRT16*, *CLIP1*, *UNC13C*, *RNF20*, *GIGYF2*, *EP400*, *TPRN*	0.003907	0.102373
Up seqfeature	Compositionally biased region: Poly-Gln	10	*ATXN2*, *CCNK*, *AR*, *ATXN3*, *KCNC3*, *IRF2BPL*, *HTT*, *KIAA0586*, *TBP*, *EP400*	1.95 × 10^−5^	4.58 × 10^−5^
Up keywords	Mental retardation	10	*IRX5*, *MAP2K1*, *WDR62*, *USP9X*, *SETBP1*, *MED12*, *DOCK8*, *CTNNB1*, *AHDC1*, *BCAP31*	6.05 × 10^−4^	0.007916
Goterm mf direct	GO:0003682~chromatin binding	10	*TFAM*, *AR*, *NOC3L*, *ARID3A*, *MED12*, *ATAD2*, *MBD2*, *RNF20*, *EP400*, *KDM6B*	0.022881	0.147425
Up keywords	Triplet repeat expansion	6	*ATXN2*, *AR*, *ATXN3*, *IRF2BPL*, *HTT*, *TBP*	3.76 × 10^−5^	2.46 × 10^−4^

FDR: false discover rate.

## Supplementary Materials

Supplementary materials can be found at www.mdpi.com/1422-0067/18/1/81/s1.
