# Supplementary material for: Somatic Genetic Variation in Solid Pseudopapillary Tumor of the Pancreas by Whole Exome Sequencing
Source: Int J Mol Sci. 2017 Jan 3;18(1):81. doi: 10.3390/ijms18010081 (PMC5297715; doi:10.3390/ijms18010081)
Supplement: Supplementary file 1 [file ijms-18-00081-s001.pdf]

Supplementary Materials: Somatic Genetic Variation in Solid Pseudopapillary Tumor of the Pancreas by Whole Exome Sequencing

Meng Guo, Guopei Luo, Kaizhou Jin, Jiang Long, He Cheng, Yu Lu, Zhengshi Wang, Chao Yang, Jin Xu, Quanxing Ni, Xianjun Yu and Chen Liu

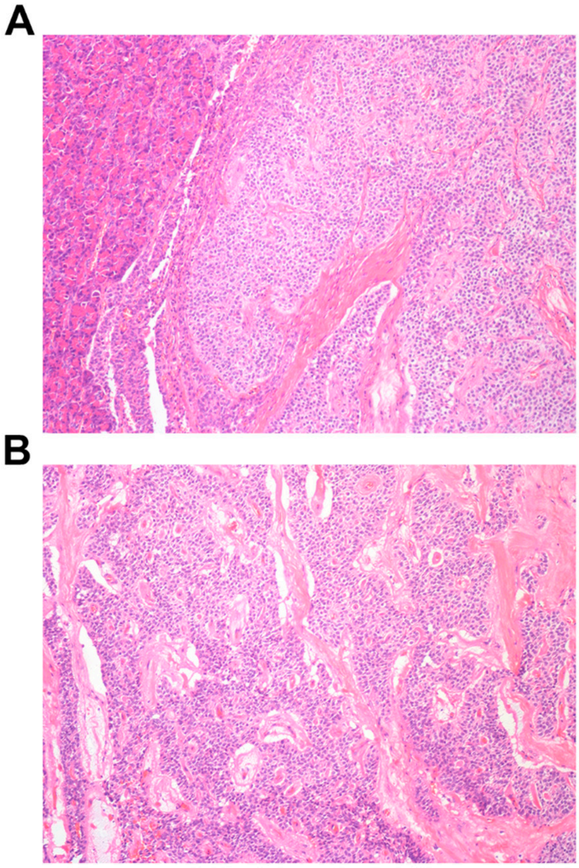

Figure S1. The solid tumor was extensively degenerated compared to normal tissue (**A**, Left half: normal tissue; Right half: SPT tissue, HE staining 100×), and the cells attached loosely around the angiogenic regions to form the pseudopapillary structure, with a lack of adenoid structure (**B**, HE staining 100×).

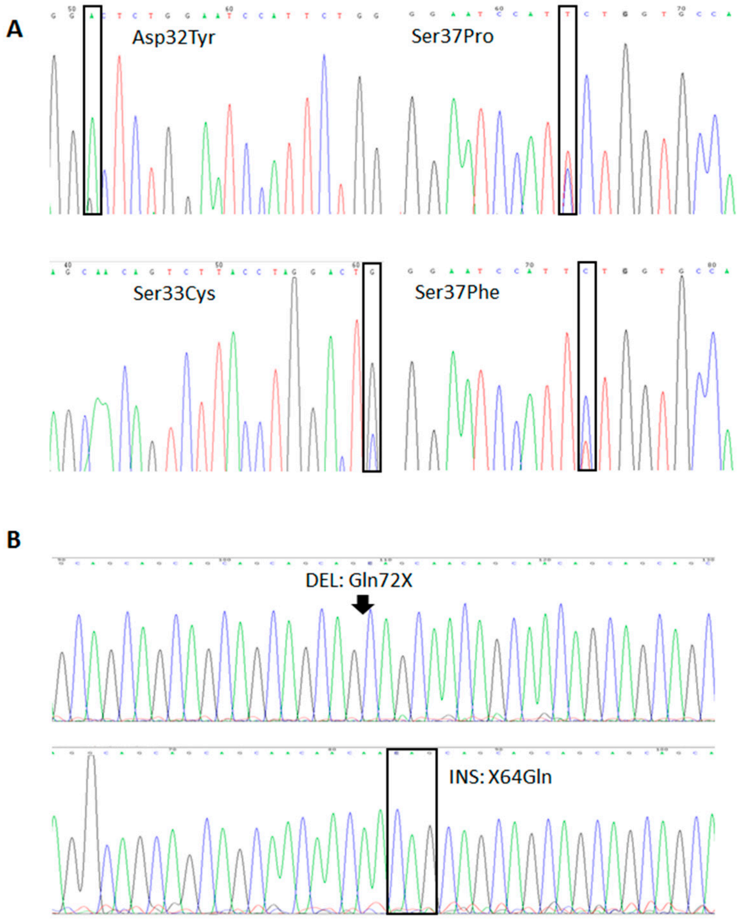

Figure S2. Representative variations of frequent SNPs in *CTNNB1* (**A**); and indels in *TBP* (TATA-Box binding protein) (**B**) were validated by Sanger sequencing (Green line: Adenine; Red line: Thymine; Black line: Guanine; Blue line: Cytosine).
